# Heart Rate and Heart Rate Variability Change with Sleep Stage in Dairy Cows

**DOI:** 10.3390/ani11072095

**Published:** 2021-07-14

**Authors:** Laura B. Hunter, Marie J. Haskell, Fritha M. Langford, Cheryl O’Connor, James R. Webster, Kevin J. Stafford

**Affiliations:** 1 Animal Behaviour and Welfare, Ethical Agriculture, AgResearch Ltd. Ruakura Research Centre, Hamilton 3214, New Zealand; Cheryl.OConnor@agresearch.co.nz (C.O.); Jim.Webster@agresearch.co.nz (J.R.W.); 2 Animal Behaviour and Welfare, Animal and Veterinary Sciences, Scotland’s Rural College (SRUC), Edinburgh EH9 3JG, UK; Marie.Haskell@sruc.ac.uk (M.J.H.); Fritha.Langford@sruc.ac.uk (F.M.L.); 3 School of Agriculture and Environment, Massey University, Palmerston North 4474, New Zealand; K.J.Stafford@massey.ac.nz

**Keywords:** dairy cows, heart rate, sleep, heart rate variability, polysomnography

## Abstract

**Simple Summary:**

The amount of sleep acquired and changes to patterns of sleep could be a useful tool to assess cow welfare, particularly in response to changes or stressors in their environment. However, the current most accurate method to assess sleep, polysomnography (PSG), is difficult and time consuming. In humans, heart rate (HR) and variability in time between heart beats (HRV) can be used to identify sleep stages, and this could be a useful alternative to investigate sleep in cows. We compared measures of HR and HRV with PSG in two groups of dairy cows in different environments and investigated the effects of lying posture on these measures. We found that HR decreased with deepening sleep stages in both groups of cows, that rapid eye movement sleep (REM) was associated with higher HRV and that HR and HRV also changed with different lying postures. The patterns of differences between sleep stages were similar between the two groups of cows. Our results suggest that HR and HRV change with sleep stages in cows and that these measures could be a useful, and more easily applied, method of assessing sleep stages in dairy cows.

**Abstract:**

Changes to the amount and patterns of sleep stages could be a useful tool to assess the effects of stress or changes to the environment in animal welfare research. However, the gold standard method, polysomnography PSG, is difficult to use with large animals such as dairy cows. Heart rate (HR) and heart rate variability (HRV) can be used to predict sleep stages in humans and could be useful as an easier method to identify sleep stages in cows. We compared the mean HR and HRV and lying posture of dairy cows at pasture and when housed, with sleep stages identified through PSG. HR and HRV were higher when cows were moving their heads or when lying flat on their side. Overall, mean HR decreased with depth of sleep. There was more variability in time between successive heart beats during REM sleep, and more variability in time between heart beats when cows were awake and in REM sleep. These shifts in HR measures between sleep stages followed similar patterns despite differences in mean HR between the groups. Our results show that HR and HRV measures could be a promising alternative method to PSG for assessing sleep in dairy cows.

## 1. Introduction

Two main stages of sleep exhibited by animals are known as rapid eye movement sleep (REM) and non-REM sleep. Non-REM sleep has been associated with restorative functions in the body and brain, for example, the clearance of potentially harmful toxins produced by normal cellular function [1]. REM sleep has been associated with memory, learning and dreaming [2]. Changes to the amount and patterns of sleep stages could be used to assess animal welfare, as these aspects of sleep are known to be affected by factors such as environmental conditions, stressful occurrences during the day, pain or illness [3]. For example, after moving into an unfamiliar environment, cows were found to spend less time lying in postures associated with sleep than their baseline, which could be an indication of stress [4]. However, in dairy cows, without using neuro-electrophysiological methods, it is difficult to accurately identify sleep from wakefulness, let alone different sleep stages.

As sleep is a homeostatic function originating in the brain, the most accurate way to study it is through polysomnography (PSG), the study of multiple electrophysiological signals, namely brain activity, eye movements and muscle activity [5]. PSG can be successfully used to study sleep in calves [6] and in adult cows [7,8]; however, it is costly, the equipment is fragile, and interpretation of the signals is time-consuming [7]. Being able to identify sleep in dairy cows with other more easily applied or less invasive devices would be beneficial not only for the cow’s comfort and welfare, but also for ease of application by researchers, thus facilitating the study of the sleep of cows and opening several new avenues for investigation of the effects of sleep loss or importance of sleep for cows.

During sleep and different sleep stages, changes occur in the regulation of the mammalian autonomic nervous system (ANS) and its subdivisions, the parasympathetic (PNS) and sympathetic nervous systems (SNS), affecting many functions such as heart rate, respiration rate and body temperature [9]. Specifically, during REM sleep, there is variability in ANS activity, leading to more variability in the associated physiological functions, whereas in non-REM sleep stages, there is more activity of the PNS while SNS activity is reduced [9]. Because the ANS affects the heart, measures of heart rate (HR) and heart rate variability (HRV: the measurement of the variability in the time between successive heart beats) can be used as a way to identify activation of the PNS and SNS [10]. In humans, changes in HR and HRV have been used to accurately identify and differentiate between sleep stages [11,12]. HRV can be quantified with different methods. Time domain indices of HRV identify differences in the time between successive heart beats or inter-beat-interval (IBI) while frequency domain indices classify the signal into frequency bands [13].

The study of HRV in cow welfare to date has focussed mainly on the application of HR and HRV to identify and assess stress. HR and HRV were found to be affected by severe lameness which may cause chronic stress in cattle [14]. Calves being disbudded without local anaesthetic showed an increase in frequency domain metrics of HRV [15]. HR and HRV has also been used to identify positive interactions in dairy cows, and social licking between cows was found to reduce heart rate in receivers [16]. Body posture has been found to affect HR and HRV measures. Heart rate was lower and variability in time between heart beats was higher in cows lying down compared to when standing [17]. However, to our knowledge, investigations of HR and HRV during sleep in cows have not been done. In previous work, we have found that sleep occurs when cows are lying down, but specific lying postures could not be used reliably to identify sleep stages compared to PSG in dairy cows. Furthermore, housing conditions have been shown to affect the relationship between lying postures and sleep [18].

It is possible that HR and HRV could be used to identify sleep stages in cows. The equipment required to assess HR and HRV is less invasive and more easily applied than equipment used for PSG. Therefore, the objective of this study was to determine if HR and HRV differ between sleep stages in dairy cows, and to determine if this is repeatable between cows in different areas, housing conditions and lying postures.

## 2. Materials and Methods

### 2.1. Ethical Statement

The study was designed in accordance with the relevant guidelines and legislation in both Scotland and New Zealand (NZ) where the studies took place. Ethical approval was obtained from the UK Home Office (Project Licence P204B097E), SRUC Animal Ethics Committee (Ref. ED AE 03-2018) and Ruakura Animal Ethics committee (AE 14708) prior to the start of animal manipulations.

### 2.2. Cows and Housing

Twelve cows were recruited for this study from two locations. Six non-lactating and non-pregnant Holstein cows (age 3.9 ± 0.7) from the Acrehead unit of SRUC’s Dairy Research Centre (Dumfries, Scotland) and six, three-year-old, non-lactating, pregnant Kiwi-cross (Friesian × Jersey) cows from the DairyNZ Lye Farm (Newstead, NZ) were used. The small sample size was necessary due to the time-intensive methods required to habituate the cows to the recording devices. Non-lactating cows were selected to avoid disruptions to the cow’s sleep patterns due to fetching for milking and the risk of damage to recording devices in the milking parlour. The Scottish cows, destined to be culled from the herd due to reduced fertility, were healthy during the trial.

**Figure 1:**
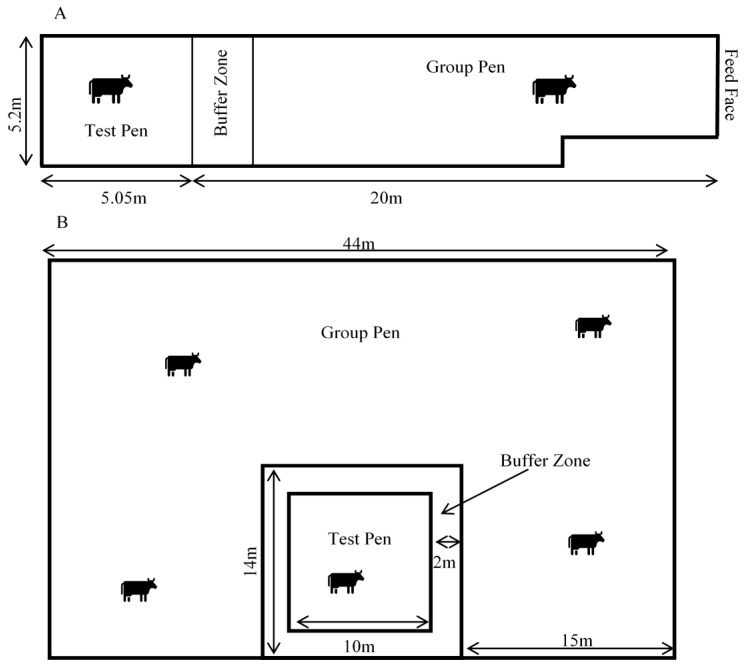
Diagrams of group and test pen design in the Scottish indoor-housed study (**A**) and in the NZ outdoor pasture study (**B**). During recordings, the test cow was moved into the test pen. When not recording, the cow was moved back into to the group pen.

The cows were managed in a large group pen and moved into a smaller adjacent ‘test’ pen individually for recording sessions (Figure 1). The Scottish cows were held on deep-bedded straw in a barn. The group pen measured 20 m × 5.2 m and test pen 5.2 m × 5.1 m. The cows were fed silage and always had access to water. The NZ cows were managed outdoors in a large paddock. They were able to graze and were provided with silage ad libitum and always had access to water. The group pen measured 44 m × 29 m and was created with live electric fencing. The 10 m × 10 m test pen was created with non-live electric fencing tape, to prevent potential interference with the electrophysiological recordings. The fencing set-up for both group and test pens could be moved around the paddock when ground conditions became wet or muddy. In both locations, a 2 m buffer zone was created between the test pen and group pen, to inhibit contact and reduce damage to recording devices from social interactions.

The cows were fitted with the recording devices and moved into the test pens individually for a maximum of 7 days. The devices and recording gear were downloaded, re-charged and re-set twice daily. Devices were removed if the cow showed signs of skin or behavioural irritation or in the case of forecasted heavy rain (NZ outdoor group).

### 2.3. Heart Rate Recording

HR and HRV were measured using a polar RS800 CX watch and Polar equine monitoring belt (Polar Electro Oy, Kempele, Finland). Patches of hair at the electrode locations were clipped and the electrodes were generously coated with ultrasound gel (Aquasonic 100 gel, Parker Laboratories, Fairfield, NJ, USA) to improve contact with the skin and signal transmission. The belt and watch were checked frequently and adjusted as needed throughout the recording. An elastic surcingle was attached over the belt to keep it tight to the skin. The clasps of the Polar belt and surcingle were padded with felt and wrapped in cohesive bandage to reduce the chance of irritation to the cows and also reduce the chance of the surcingle loosening throughout the recording. The watch was synchronized to the recording computer’s time and was programmed to record heart rate and R-R intervals which are used for HRV calculations. R-R intervals are the time (in milliseconds) from the R peak of one heartbeat to the R peak of the next heartbeat.

The data were downloaded and analysed using Polar Pro Trainer (v5.35.160) and artefacts in the R-R data were filtered and corrected using moderate filter power. Only traces containing less than 1% of identified errors were used in the analysis. Filtered data were exported and HR and HRV statistics were calculated in 30 s intervals (epochs) corresponding to the scored PSG data. Only time domain features of the HRV were calculated since frequency domain features of the HRV may not be an accurate representation of the data in such small time periods [19]. Time domain features included mean HR (in beats per minute—BPM), root mean square of successive differences of the R-R signal (RMSSD), and standard deviation of the R-R signal (SDRR) in 30 s epochs.

### 2.4. PSG Recording and Sleep Scoring

#### 2.4.1. PSG Recording Protocols

PSG were recorded as described by Hunter et al. (2021) [20]). Pre-gelled adhesive snap ECG electrodes (Natus Neurology, Ottawa, ON, Canada) were used to record four EEG, a reference (REF), patient grounding (PGND), and two EOG and two EMG channels from the cows. Lead wires were snapped on, bundled down the neck and plugged into the Embletta MPR PG + St proxy PSG recording device (Embla Systems, Ottawa, ON, Canada). The device was placed in a padded plastic box within a pouch sewn to the elastic surcingle covering the HR monitor belt. The device was programmed, data were downloaded, and traces processed and scored using RemLogic 3.4.3 software (Embla Systems, Ottawa, ON, Canada). Good quality recordings, which had a minimum of one complete EEG, EOG and EMG trace each with good impedance (1–14kHz), minimal artefacts and with good quality corresponding HR traces were used in the analysis. Recordings lasted a maximum of 10 h due to device memory limitations.

#### 2.4.2. Sleep Scoring

The good quality traces were scored according to criteria developed from a combination of previous cow sleep EEG studies [6,7,21] as well as human sleep scoring criteria [22]. Five stages of sleep and wakefulness were scored: Awake (W), REM (R) and Non-REM (which was subdivided into 3 stages, light N1 and N2, and deep N3). Rumination was also scored from the PSG as substantial artefacts due to jaw muscle movements when chewing obscured the actual signals of the traces and it was impossible to tell what stage the cow was in during that time. Intra-observer accuracy was calculated using “irr” package [23] in R (v4.0.5) using Cohen’s Kappa with κ = 0.83 and overall agreement of 89.4% indicating good agreement [24].

#### 2.4.3. Lying Postures

Lying postures were identified from video recordings made from four surveillance cameras equipped with automatic infra-red night vision capability (Geovision monitoring system, Viewlog, GeoVision Inc., Taiwan (Scottish Cows)) and Vivotek ND9541P H.265 NVR (Vivotek Inc., Taiwan) (NZ Cows)). Lying postures, with head held up above the point of the shoulder (UP), head held below the shoulder (HL), head resting on the ground to the front (HF), neck turned with head resting on flank or “tucked” (T), lateral lying or “flat out” (FO) and moving (MV) as well as not scored (NS) (Table 1), were scored instantaneously every 30 s corresponding to the start of the PSG and HR epochs. Intra-observer reliability was conducted in R (v4.0.5) [25] using the Cohen’s kappa in the ‘irr’ package [23] and the kappa statistic was κ = 0.95 demonstrating a high level of agreement [24].

### 2.5. Data Analysis

Scored sleep stages, lying postures and heart rate data were aligned by time stamps. In cows, sleep occurs when lying down [21], therefore only epochs identified as lying were included in the analysis. Epochs with posture ‘not scored’ (NS) due to observer inability to accurately observe behaviour or other extraneous circumstances were also removed from the dataset. As the stages of sleep or wakefulness could not be determined while ruminating, these epochs were also removed.

We fitted a mixed-effects model to determine if the cow’s HR changed by sleep stage using the ‘lme4′ [26] and ‘lmertest’ [27] packages in R (v4.0.5) [25]. The fixed effects were study (country), sleep stage and their interaction. We included recording day nested within individual cow ID as random factors. We then used the same model with each of the remaining variables, and RMSSD and SDRR as the response variables. Using the ‘predictmeans’ [28] package we calculated the predicted means, standard error of the means (SEM) and least significant differences (LSD).

We then re-ran the same models, now including the cow’s lying posture as a fixed effect with interaction with study and calculated predicted means of cow’s HR and HRV measures by lying posture and study.

## 3. Results

Overall, with rumination, standing, and unscored lying behaviour removed, 1968 epochs totaling 16.4 h of good quality data were obtained from 10 cows in 29 recordings days. Data from one Scottish and one NZ cow were removed as they each had only one limited good quality recording that did not contain any lying periods. The data set was skewed towards more time in the awake (W) state, as 629 epochs were scored as W, 315 epochs in N1, 593 epochs in N2, 197 epochs in N3 and finally 234 epochs in REM (Table 2).

### 3.1. Lying Posture

**Figure 2:**
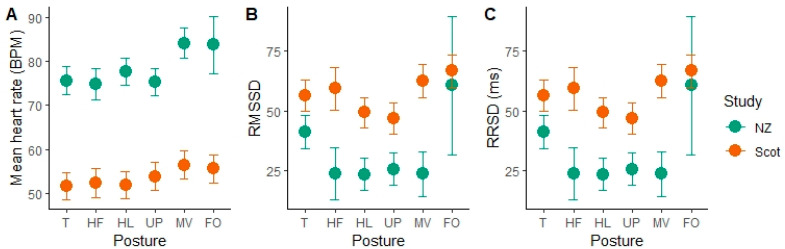
Plots of predicted means with error bars indicating standard error of the means for the mean heart rate in beats per minute (bpm) (**A**), the RMSSD (**B**) and SDRR (**C**) by lying postures (Tucked (T), head resting front (HF), head low (HL), head up (UP), moving (MV) and flat out (FO)) for the New Zealand group (NZ), Scottish group (Scot). Figure produced in R v4.0.5 using ggplot2 package (https://cran.r-project.org/web/packages/ggplot2/index.html, accessed on 3 May 2021).

The HR and HRV parameters changed with specific body posture while lying. In the Scottish cows, moving and lying flat out postures resulted in significantly higher mean heart rate (MV = 56.43 ± 3.17 bpm, FO = 55.53 ± 3.15 bpm) than all other postures (lying with the head up, or low, or resting on the ground or with the head tucked) (Figure 2). Flat out lying was rare in the NZ data, with only one epoch over all observations. Moving was also associated with a higher mean HR in the NZ group (84.22 ± 3.41 bpm). In the NZ cows, tucked posture was also associated with significantly higher RMSSD values than the head low, head up and moving postures, indicating more variability in the time between successive heart beats. Similar results were found with RMSSD in the Scottish group, who had higher RMSSD in T compared to HL (*p* = 0.007, df = 1936, t = 2.7), UP (*p* = 0.0017, df = 1936, t = 3.15) and lower compared to FO (*p* = 0.0035, df = 1936, t = −2.92). We also found a significant effect of sleep stage and its interaction with study location on the HR and HRV parameters. Table 2 shows the means for the different sleep stages.

### 3.2. Mean HR

After accounting for variation between cows and study days, we found a large effect of study group on mean HR. Mean HR was around 20 BPM lower in the Scottish cows than the NZ cows. After accounting for this variation, significant differences between sleep stages were evident. In both the indoor-housed Scottish group and outdoor-managed NZ cows, mean heart rate was significantly slower in the REM sleep stage compared to awake (W) (NZ: *p* < 0.001, df = 1934, t = 5.51) (Scottish: *p* < 0.001, df = 1934, t = 12.16). In the Scottish group, N2 and N3 stages were not significantly different from one another (*p* = 0.09, df = 1934, t = 1.68), but all others (W, N1, R) were. In the NZ group, W and N1 were not different from each other (*p* = 0.46, df = 1934, t = −0.74), and neither were N3 and REM (*p* = 0.89, df = 1934, t = 0.14). Overall, heart rate declined successively from W to N1 and then to N2, while N3 and REM were significantly lower than the other sleep stages.

### 3.3. RMSSD-Variability between Successive Heart Beats

**Figure 3:**
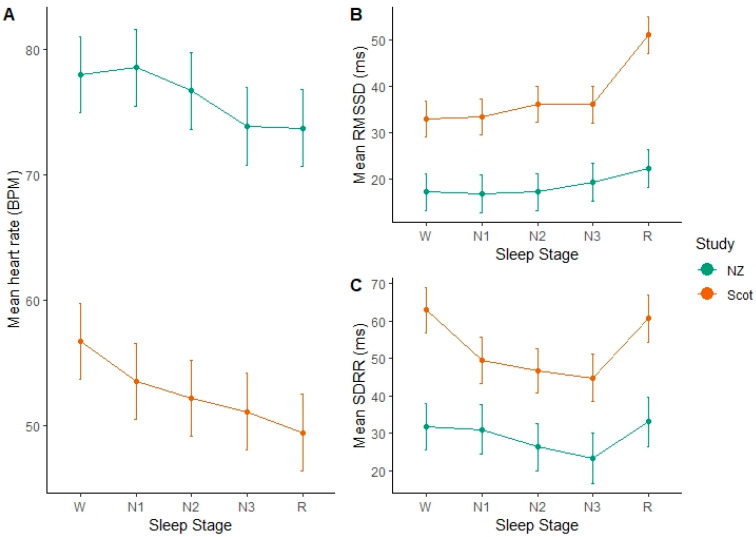
Plots of predicted means with error bars indicating standard error of the mean in each sleep stage for the NZ and Scotland groups for mean HR (**A**), RMSSD (**B**) and SDRR (**C**)). Figure produced in R v4.0.5 using ggplot2 package (https://cran.r-project.org/web/packages/ggplot2/index.html, accessed on 3 May 2021).

As heart rates were significantly different between the study groups, it is unsurprising that they also had a significant effect of study on the RMSSD (Figure 3). As the mean heart rate was lower in the Scottish group, their RMSSD was 15–30 ms higher than the NZ group, indicating longer inter-beat intervals (Table 3). Accounting for the random effects, we found significantly higher RMSSD values during REM sleep epochs, indicating more variability in the time between successive heart beats in this stage. In the NZ group, the RMSSD during REM sleep was significantly higher than W (*p* = 0.0061, df = 1937, t = −2.74), N1 (*p* = 0.01, df = 1937, t = −2.57) and N2 (*p* = 0.0056, df = 1937, t = −2.78) but not significantly different from N3 (*p* = 0.18). In the Scottish group, RMSSD during REM sleep was highly significantly different from all other sleep stages, which did not differ greatly from one another. However, N2 did differ significantly from W (*p* = 0.002, df = 1937, t = −3.09) and N1 (*p* = 0.028, df = 1937, t = −1.554). Overall, the time between successive heart beats during REM sleep was significantly more variable than the other stages. N3 was more variable than W but not compared to the other NREM stages (N1 & N2).

### 3.4. SDRR-Total Variability of Time between Heart Beats

There were differences between the groups, but this was not as wide as for the other variables. SDRR was higher for the awake and REM stages compared to the other stages, indicating that there was higher variability in the overall time between heart beats for these stages. In the NZ cows, SDRR during REM sleep was significantly higher than N3 (*p* = 0.0288, df = 1947, t = −2.1882), but not the other stages. N3 was significantly lower than W (*p* = 0.0337, df = 1947, t = 2.1254), however N3 was not significantly different from the other stages. In the Scottish group, SDRR was not significantly different between W and REM (*p* = 0.4422, df = 1947, t = 0.7687), but these stages were significantly higher than N1, N2 and N3 that were not significantly different from one another.

## 4. Discussion

Our results show that cardiac outputs could be useful in assessing sleep stages in dairy cows. However, we found major differences in mean HR between the two groups of cows, which may be due to different cow characteristics. Understandably, despite replication in data collection methods, there were marked differences in the housing, breed, size, physiological stage, and diet of the cows in each study location. The NZ cows were all in late pregnancy, whereas the Scottish cows were non-pregnant and non-lactating. Late pregnant heifers and cows have been found to have higher mean HR than earlier on in pregnancy [29]. While there was not a particularly large difference in cow age, there was a difference between cow size and breed. The Scottish cows were very large Holstein cows, and the NZ cows were much smaller being Jersey-Holstein crosses (Kiwi-cross). Other studies have found significant differences in HR and HRV measures between different breeds (Brown Swiss and Simmental) when standing, lying and milking [30]. Body size is also known to affect HR and HRV, and a decrease in HR was found with increasing weight in horses and ponies [31]. The Scottish recordings were made indoors in spring/summer months with a daytime temperature average of 15.2 °C (range 8–22 °C) and overnight temperature of 11.2 °C (range 5–22 °C). The NZ recordings were made outdoors over winter with average an average daytime temperature of 10.2 °C (range 2–18 °C) and overnight average of 8.4 °C (range 2–14 °C). Seasonal thermal stress has been found to affect behaviour, stress and immune response in dairy cows [32,33], and increasing temperature humidity index has been associated with decreased HRV measures in sheep and goats [34]. Although the cows in the winter conditions in NZ had higher HR and lower HRV than cows in summer conditions in Scotland, the environmental conditions could have affected the HR and HRV activity in this study.

Importantly, despite these group differences, we found that HR and HRV changes with sleep stages in both groups and clearly, Figure 2 shows that the differences are in the same direction. These results indicate that the patterns of the changes in HR and HRV measures between the sleep stages are stable and as such these measures could be used with all cows, although further research is needed to assess if these patterns are also observed in lactating cows and cows in other stages of pregnancy.

Surprisingly, we found that mean HR during REM sleep was lower than when awake and in the lighter NREM sleep stages (N1 and N2). This is different than results in humans, where HR has been found to decrease with the progression of NREM sleep stages, and speeds up again in REM sleep [35]. However, similar results with an overall lower heart rate during REM sleep were also found in dogs [36]. Despite the lower mean HR, HRV measures of RMSSD and SDRR were higher in REM sleep, indicating more variation between heart beats. This observation is similar to that shown for HRV patterns during human sleep, where HRV tends to be more variable when awake and in REM sleep than during N3 and other NREM sleep stages [37]. Mean HR and RMSSD may be useful to distinguish between awake (W) and REM stages; however, they are not particularly useful to distinguish between NREM stages (N1, N2, N3). SSDRR was useful to identify N3 stages in both groups as it was significantly lower. These patterns of differences in sleep stage could be useful in future applications to predict sleep stage of dairy cows, particularly if prior to recording, a lying awake baseline could be specified. Then sleep stages could be identified or predicted from differences from that baseline.

A previous study has found that body position was associated with difference in HR and HRV measures in cows [17]; however, they did not specify body posture while lying and were unable to identify awareness levels. We found that the specific posture that cows adopted during lying affected their HR and HRV, and in particular that epochs identified as being in the flat out (lateral lying) posture and epochs with the head moving resulted in higher HR and more variability in the HRV. As moving is a physically active behaviour, this activity may have had a carry-over effect on the heart rate for an extended period. Therefore, an epoch in which the cow moved her head at the start may have higher HR across that epoch and into the next. Flat out posture was rarely observed in the NZ group, and only scored once, and even then, was only observed as a transition behaviour between other postures. In the Scottish group, flat out postures were far more commonly observed, and most often occurred while the cow was in N2 sleep as well as awake and in N1. It is unclear if the increased HR in this posture was due to the position of the body which could have facilitated a faster movement of blood, or because most epochs scored as flat out happened to occur in sleep stages that had higher heart rates. In the NZ group almost all REM sleep occurred in the tucked posture. The tucked posture was found to have significantly higher RMSSD; however, since REM sleep was also found to have higher RMSSD it is likely that the effect of the posture on the HRV measure was more likely due to the sleep stage in this case.

The intra-observer reliability for sleep scoring was 89%, which according to inter-scorer reliability in human sleep studies is very good [38,39]. However, there is still some possibility that the 11% uncertainty in sleep scoring was a contributing factor to the variability of the HR and HRV measures within sleep stages. Additionally, we analysed the HR data in 30 s epochs, specifically to correspond to sleep scoring. The 30 s epoch is a standard practice in scoring sleep stages from PSG, as it corresponds well to the structure of human sleep, containing fewer stage shifts than longer epochs which would be more likely to contain many stage shifts [40]. Despite shorter epoch length, some mid-epoch stage shifts could still have occurred. In these instances, although the PSG was scored one way, the HR measures could have reflected another stage, and this could also be another source of variability in the HR and HRV measures within sleep stage.

Similarly, the HR and HRV data may have also been influenced by the short epoch windows. Typically, human HRV measures are conducted in 5 min increments, although ultra-short windows such as 30 s windows have been found to be acceptable for the assessment of HRV at rest in humans [41]. Bouts of cow sleep stages can typically be quite short [7,21] and thus multiple stage shifts would be captured in a longer epoch length of 5 min. This was a major reason for choosing to analyse the HR and HRV in ultra-short windows. However, some have questioned the accuracy of windows shorter than 2-min for the analysis of HRV in human athletes [19]. RMSSD measurements in even shorter 10 s windows were also found to correspond well to standard longer intervals in humans, but SDRR did not [42]. Therefore, the short time window selection could have affected the accuracy of the cow HRV RMSSD and SDRR measurements. HR and HRV may be useful for the assessment of sleep stages in dairy cows, however, further investigation into the validity of ultra-short HRV measures in dairy cows and additional validation with PSG is needed.

## 5. Conclusions

We have shown that sleep stage is associated with changes in HR and HRV in dairy cows. Mean HR was significantly lower in the indoor-housed, non-pregnant, and non-lactating cows compared to pregnant, dry, outdoor managed cows. We also found that mean HR decreased with sleep depth, SDRR was more variable in awake and REM states, and RMSSD was significantly higher in REM sleep than the other stages. These results indicate that HR and HRV could be a useful measure for the future identification of sleep stages in dairy cows using less invasive devices than PSG, making sleep research for animal welfare more accessible.

## Figures and Tables

**Table 1:** Behavioural ethogram for scoring lying postures in dairy cows, including head positions and photographs from surveillance videos.

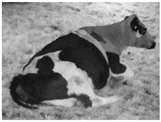	Head Up (UP) Lying sternally recumbent with head held up
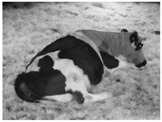	Head Low (HL) Lying sternally recumbent with head held low
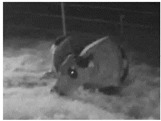	Head Resting Front (HF) Lying sternally recumbent with the head and or neck resting on the ground
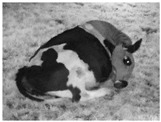	Tucked (T) Lying sternally recumbent with head turned and resting on the flank
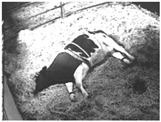	Flat-out (FO) Lying laterally with legs extended and head and neck resting on the ground

**Table 2:** Count of the total number of epochs of data in each posture by sleep stage (tucked (T), head resting front (HF), head low (HL), head up (UP), moving (MV) and flat out (FO)) and study country.

	NZ							Scot						
	T	HF	HL	UP	MV	FO	Total	T	HF	HL	UP	MV	FO	Total
W	24	2	67	116	14	1	224	42	4	133	157	49	20	405
N1	4	2	34	58	3	0	101	20	1	103	52	13	25	214
N2	9	4	61	120	0	0	194	51	13	217	46	6	66	399
N3	8	2	21	47	0	0	78	17	1	72	15	3	11	119
R	90	0	0	1	0	0	91	114	1	4	3	1	20	143
Total by Posture	135	10	183	342	17	1	688	244	20	529	273	72	142	1280

**Table 3:** Table of predicted means ± standard error of the HR mean, RMSSD and SDRR for each of the stages, awake (W), Non-REM: N1–N3 and REM sleep overall data and by study group in NZ and Scotland (SC).

Sleep Stage	W	N1	N2	N3	REM
HR Mean					
Predicted Mean NZ	78.03 ^A^ ± 3.05	78.57 ^A^ ± 3.09	76.72 ^B^ ± 3.06	73.90 ^C^ ± 3.10	73.78 ^C^ ± 3.09
Predicted Mean SC	56.77 ^D^ ± 3.04	53.56 ^E^ ± 3.05	52.20 ^F^ ± 3.04	51.14 ^F^ ± 3.08	49.48 ^G^ ± 3.07
RMSSD					
Predicted Mean NZ	17.15 ^A^ ± 3.97	16.80 ^A^ ± 4.12	17.14 ^A^ ± 3.99	19.22 ^AB^ ± 4.18	22.14 ^BC^ ± 4.14
Predicted Mean SC	32.92 ^CD^ ± 3.82	33.41 ^DE^ ± 3.88	36.03 ^F^ ± 3.83	35.96 ^EF^ ± 3.98	50.96 ^G^ ± 3.94
SDRR					
Predicted Mean NZ	31.74 ^AB^ ± 6.22	30.95 ^ABC^ ± 6.60	26.30 ^AC^ ± 6.27	23.32 ^C^ ± 6.75	33.08 ^ABD^ ± 6.63
Predicted Mean SC	62.93 ^E^ ± 6.03	49.61 ^D^ ± 6.19	46.69 ^BD^ ± 6.04	44.83 ^BD^ ± 6.42	60.71 ^E^ ± 6.34

^A–G^ Means within each HR/HRV measure without a common superscripted letter are significantly different at *p* < 0.05.

## Data Availability

The data that support the findings of this study are available from the corresponding author, L.B.H., upon reasonable request.
